# Evaluation of the Effectiveness of Eugenol and MS-222 as Anesthetics in Zebrafish in Repeated Exposures and Post-Anesthesia Behaviour

**DOI:** 10.3390/ani14162418

**Published:** 2024-08-21

**Authors:** Nahúm Ayala-Soldado, Rafael Mora-Medina, Ana María Molina-López, Antonio Jesús Lora-Benítez, Rosario Moyano-Salvago

**Affiliations:** Department of Anatomy and Comparative Pathology and Toxicology, UIC Zoonosis y Enfermedades Emergentes ENZOEM, Faculty of Veterinary Medicine, Campus de Rabanales, University of Córdoba, Darwin Building, 14071 Córdoba, Spain; nahum.ayala@uco.es (N.A.-S.); v12lobea@uco.es (A.J.L.-B.); r.moyano@uco.es (R.M.-S.)

**Keywords:** anesthesia, eugenol, induction time, MS-222, post-anesthesia behaviour, recovery time

## Abstract

**Simple Summary:**

In this study, we compared the effectiveness of two anesthetics, eugenol and tricaine methanesulfonate (MS-222), in consecutive administrations on zebrafish. We evaluated zebrafish behaviour after repeated anesthesia. Eugenol induced anesthesia more quickly than MS-222 but had longer recovery times. Additionally, swimming frequency decreased after eugenol anesthesia. The buffered version of MS-222 was more effective than the non-buffered one. We recommend using buffered MS-222 for studies requiring repeated, brief-duration anesthesia.

**Abstract:**

The increasing use of the zebrafish (*Danio rerio*) in scientific experiments has made it necessary to implement anesthesia protocols guaranteeing minimum pain and suffering for these animals and ensuring the reliability of the results obtained from their research. Therefore, we aimed to compare the effectiveness of two anesthetics, eugenol and MS-222, in consecutive administrations and evaluate the zebrafish behaviour after repeated anesthesia. Thus, several zebrafish were anaesthetized with eugenol, MS-222, and buffered MS-222 three times repeatedly with a 24-h interval between each exposure. The induction and recovery periods were also timed. Their swimming frequency was determined after each exposure to assess their behaviour after the anesthesia. Anesthesia induction was quicker with eugenol compared to MS-222. However, eugenol presented longer recovery times, which were prolonged after each exposure. Also, the swimming frequency was reduced after each anesthesia with eugenol. The buffered version of MS-222 was more efficacious than the non-buffered one. Both versions of MS-222 did not affect the swimming frequency. Based on these findings, we recommend the utilization of MS-222 buffered rather than eugenol when repeated, brief-duration anesthesia is necessitated for a study.

## 1. Introduction

The use of the zebrafish (*Danio rerio*) in research has considerably increased in recent years, and it has shown itself to be a useful animal in diverse fields, such as biomedicines or environmental sciences [1,2,3]. In this context, the care and use of animals for scientific purposes are governed by the internationally established principles of replacement, reduction, and refinement [4]. Reducing the animal’s suffering by means of the refinement of experimental procedures is essential not only for ethical reasons but also to ensure the quality of the investigations [5,6]. That is why experiments with animals that may cause them pain or anxiety must be performed under sedation, analgesia, or anesthesia [7].

The correct use of anesthetic is fundamental for minimizing the pain and the variability of the data obtained in the wide variety of procedures carried out repeatedly and routinely on zebrafish [8,9]. Anesthetics in fish suppress pain and have a calming effect, followed by a loss of balance, mobility, and consciousness [10]. However, while anesthesia is induced, there is an excitement phase where there is a higher risk of physical injury or escape/jump from the container or aquarium [11]. This depression of the nervous system reduces their voluntary movements and sensorial perception during the experiment [10,12]. The different anesthesia stages in fish can be categorized as follows: (0) normal, (I) light sedation, (II) deep sedation, (III) light narcosis/excitement phase, (IV) deep narcosis, (V) light anesthesia, (VI) surgical anesthesia and (VII) overdose [11,13]. The main monitoring parameters on the anesthesia depth are the opercular beat rate, immobility, or the response to tail fin pinch. The opercular beat rate decreases as zebrafish progress through each stage of anesthesia. Regarding immobility, in the initial stages of anesthesia, zebrafish may exhibit erratic swimming that will eventually diminish as they become anaesthetized. Finally, if a zebrafish is not deeply anaesthetized, it will dart off in response to a painful stimulus, such as a tail fin pinch [9].

Currently, a considerable number of anesthetics are available for use in animal research. The selection of a product not only depends on requirements such as availability, price, ease of use, not having an influence on research results, the safety of its handler or the work’s nature but also its effectiveness, which depends on biological (species, size, weight or the reproductive development state) and environmental factors (temperature, pH and water salinity) [11,14,15].

Tricaine methanesulfonate (MS-222) (3-aminobenzoic ethyl ester methanesulfonate) belongs to the local anesthetic family and is a relatively expensive compound [16]. It is an isomer of benzocaine with an additional sulfonate radical that makes it more soluble. This increase in solubility also implies a marked acidic character in the solutions; therefore, a buffer solution, such as sodium bicarbonate, is recommended when employing it [15,17]. Despite being the anesthetic most used in fish [18], situations of aversion to this drug have been reported [11,16,19]. However, the aversion is still debatable [20], and with greater refinement, it would not occur.

Eugenol (4-allyl-2-methoxyphenol) is the principal active component of clove oil, a natural product obtained from the distillation of the clove tree flower (*Syzygium aromaticum*) [21]. Although it is a widely used anesthetic in fish research, it is known that eugenol can modulate plasma cortisol levels [22,23], and it could be potentially toxic to the fish brain [24]. It is a highly lipophilic substance and is quickly absorbed through the gills and the skin [25]. At room temperature (25 °C), the solubility of eugenol is very limited; therefore, it should be dissolved in ethanol to be used [9,26].

Clearly, there are few studies that address the efficacy of anesthetic agents in zebrafish after repeated exposures, but even fewer that evaluate the post-anesthesia behaviour of the animals after several hours. The necessity for repeated administration of anesthesia to zebrafish may be required in certain experimental protocols, such as successive intraperitoneal inoculation of drugs, successive oral gavage, repeated blood extraction, or the creation of zebrafish with a specific pathology, making its investigation presently imperative. These procedures can extend for several minutes, and prolonged, repeated exposure on several occasions can lead to adverse effects such as neurotoxicity, cumulative toxicity, or even mortality. For this purpose, we aimed to compare the efficacy of eugenol and MS-222 as anesthetics in zebrafish after repeated exposure. Both the induction time of light anesthesia (stage V) and the recovery time for both drugs, after three successive exposures, were determined. In addition, recovery behaviour was evaluated by counting the number of crossings that the animals made through the aquarium 23 h after anesthesia.

## 2. Materials and Methods

### 2.1. Animals and Husbandry

The study was carried out at Servicio de Animales de Experimentación (SAEX) of the University of Córdoba, where the zebrafish were provided. 60 zebrafish wild-type AB (30 males and 30 females) of around 16 weeks were employed. All of them were clinically healthy and free from malformations. Regarding this, the zebrafish did not present any clinical signs in the visual inspection, and their swimming patterns and behaviour were free of any abnormalities. The regular health monitoring protocol in the experimental center ensured the absence, among others, of *Cytophaga* sp., *Edwardsiella ictaluri*, *Flexibacter maritimus*, *Mycobacterium* sp., *Streptococcus iniae*, ISKNV infectious spleen and kidney necrosis virus, ZFERV and ZFERV-2 (zebrafish retroviruses), *Ichthyophthirius multifiliis*, *Pleistophora hyphessobryconis*, and *Pseudoloma neurophila*. Determining the DNA or RNA of these agents was conducted by PCR or RT-PCR according to each case.

The animals were kept under a photoperiod of 16 h of light and 8 of darkness, with a constant temperature in the laboratory of 25 ± 2 °C. They were maintained in a water recirculation system (Tecniplast, Buguggiate, Italy) with filtering and germicidal ultraviolet radiation. The system itself controlled the parameters of temperature (26 ± 1 °C), pH (7–8), and conductivity (between 300 and 500 µS/cm) of the water. The hardness, ammonia (NH_3_/NH_4_^+^), nitrite (NO_2_^−^), and nitrate (NO_3_^−^) were measured by the Total Hardness Test (Ref: 1.14652; Sigma Aldrich, St. Louis, MO, USA), Ammonium Test (Ref: 1.08024; Sigma Aldrich), Nitrite Test (Ref: 1.08025; Sigma Aldrich), and Nitrate Test in freshwater (Ref: 1.11169; Sigma Aldrich). All these tests were applied following the manufacturer’s instructions. The levels of hardness, ammonia, nitrite, and nitrate were 50–250 mg of CaCO_3_, <0.1 mg/L, <0.3 mg/L, and <25 mg/L, respectively. The water in the system was already stable, but continuous monitoring was carried out. In the laboratory, hardness tests were carried out every two weeks, and nitrogen residue tests were carried out weekly. The animals were fed three times a day with a combination of dry feed (Tropical Supervit Premium^®^) (Tropical, Chorzów, Poland) and frozen *Artemia* spp. (Ocean Nutrition, Essen, Belgium). The fish were kept in groups of five inside this system at a density of one fish per litre of water for 14 days prior to the experiment. This low density was chosen due to the experimental design. In addition, some OECD guides indicate it as appropriate [27]. For each group, males and females were mixed in a tank with a ratio of 2/3 and 3/2. We decided to use these fish densities to ensure the water quality and the handling capacity of the facilities.

All procedures complied with the instructions of the Animal Experimentation Committee at Córdoba University (04/05/2018/076).

### 2.2. Experimental Design and Anesthetics Solution

The anesthetics employed were 99.9% pure MS-222 (CAS: 886–86-2, Ref: A5040, Sigma Aldrich) and 99% pure eugenol (CAS: 97–53-0, Ref: W246719, Sigma Aldrich). To buffer the MS-222 solution, bicarbonate (NaHCO_3_) was applied at double the concentration tested of MS-222 [28]. To enable the solubility of eugenol, the latter was previously dissolved as a 10% solution in 96% pure ethanol (C_2_H_6_O) [26].

The study concentrations established for eugenol were 85 mg/L and for MS-222 150 mg/L, both for the buffered and non-buffered solutions. Those concentrations were selected based on the bibliography available [9,15,26,29], considering the ideal characteristics of the anesthetics, namely, that they were able to induce anesthesia in under 3 min, which would be appropriate for the experimental procedures and to enable a rapid recovery [9,11]. The anesthetic solutions were made with water from the system and remade every day. The mean pH of the water system used before making the anesthetic solution and for eugenol, MS-222, and MS-222 buffered solutions were 7.3 ± 0.2, 7.2 ± 0.1, 6.7 ± 0.3, and 7.2 ± 0.2, respectively.

**Figure 1:**
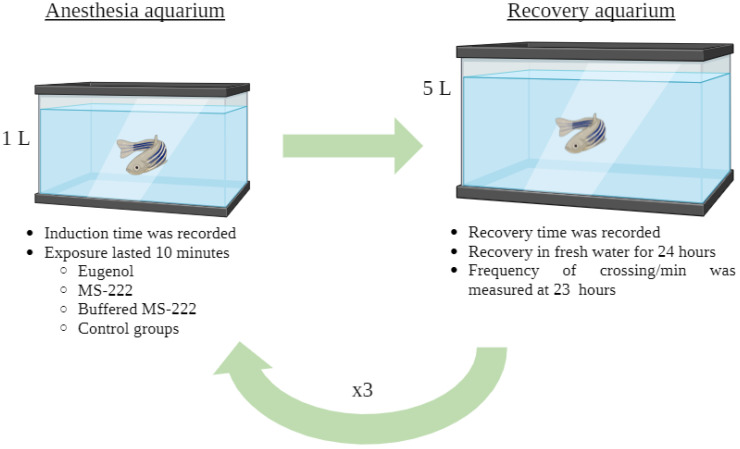
Schematic diagram of the experimental procedure.

The fish were allocated randomly in groups of 10 individuals for each concentration (5 males and 5 females). Three control groups of 10 fish each were included; two groups were exposed to the concentrations of bicarbonate (300 mg/L), ethanol (789 mg/L), and a negative control. The experimental protocol consisted of exposing and maintaining the animals repeatedly at the light anesthesia (stage V) and waiting for their total recovery from it. Each animal underwent a total of 3 exposures, with an observation interval of 24 h between them. The anesthesia was performed individually in 1 L aquariums for 10 min. Then, zebrafish were transferred into recovery aquariums. Both the anesthesia and recovery from it were recorded with a stopwatch. Regarding the control groups, the animals were placed individually in water from the system with bicarbonate, ethanol, or fresh water for 10 min, and then they were transferred to a recovery aquarium, the same as those anesthetized. It was expected that the behaviour of the zebrafish used as a control during those 10 mins would be different since the animals were awake. But it was not taken into account. Due to the requirements of the study, all the animals remained alone during this process, maintaining visual contact with other animals. A schematic diagram of the experimental procedure is shown in Figure 1. Since the toxicity of anesthetics can vary according to the daily rhythm of the zebrafish [30], all exposures were carried out at the same time of day, when the laboratory lights had been on for several hours (at a time when the fish were most active).

### 2.3. Onset and Recovery from Light Anesthesia (Stage V)

The induction time of light anesthesia (stage V) was defined as the period from when the zebrafish came into contact with the anesthetic solution until they presented a complete loss of body movement, no response to tail fin pinch with the fingers, and a diminution in their respiratory frequency [11,31]. During the 10-min duration of anesthesia, the fish were thoroughly monitored by observing the parameters mentioned earlier. Recovery time was defined as the period between transferring the fish to the recovery aquarium with fresh water up to its total recovery of balance and the restoration of the normal swimming pattern, i.e. until they could swim in the water column [9,32].

### 2.4. Post-Anesthesia Behaviour Analysis

The fish were kept for 24 h in the recovery aquarium (5 L) with oxygen values close to saturation, i.e., with an oxygenator constantly introducing oxygen. To evaluate the behaviour after anesthesia, the activity of the fish was measured approximately 23 h after the anesthesia. For this, the frequency of crossing the longitudinal line of the aquarium per minute (crossings/min) was accounted for [33]. The longitudinal line refers to the imaginary line that runs lengthwise from one end of the aquarium to the other. The frequency of crossing this line was also determined in the control groups. In order not to interfere with the results and to facilitate counting, the animals were recorded with cameras, and the data were obtained later.

### 2.5. Statistical Analysis

The statistical analysis was carried out using the Minitab Statistical Software (version 20.3, Minitab, Coventry, UK). To make the graphs, the software GraphPad Prism (version 9.4.1, GraphPad Software, Boston, MA, USA) was used. The Kolmogorov–Smirnov test and the Levene test were applied to assess data normality and variance homoscedasticity. Subsequently, a mixed linear model was fitted using the following equation:

Y = µ + A + S + E + (A × S) + (A × E) + (A × E × S) + R + ε(1)

where Y is the dependent variable (induction time, recovery time, and number of crosses/min); µ is the overall mean; A is the fixed effect of the anesthetic agent used (eugenol, MS-222, or MS-222 buffered); S is the fixed effect of the sex (male or female); E is the fixed effect of exposure (first, second, or third); R is the random effect of each zebrafish; and ε is the residual error. The Tukey test was used as a post hoc method.

In all cases, a value of *p* < 0.05 was considered statistically significant.

## 3. Results

### 3.1. Onset of Light Anesthesia (Stage V)

**Figure 2:**
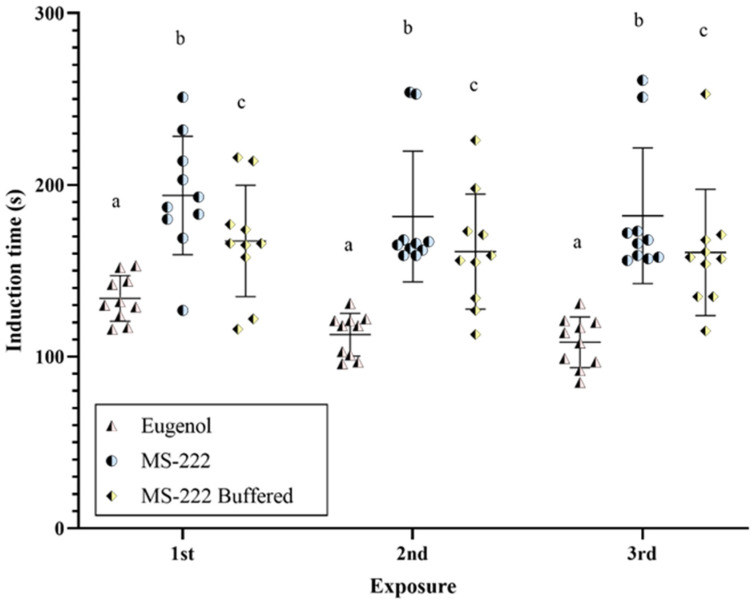
Induction times for each zebrafish exposed to each anesthetic agent after three exposures. The lines and bars represent the mean and the standard deviation, respectively. Different letters indicate statistically significant differences (*p* < 0.05).

The induction times for each anesthetic agent in repeated exposures are shown for each zebrafish individually in Figure 2. Regarding statistical analysis, the random effect of each zebrafish was not significant (*p* = 0.46). The fixed factors of sex (*p* = 0.42) and exposure (*p* = 0.13) were also not significant. However, the type of anesthetic agent used had a significant effect (*p* < 0.05) on the induction time. Specifically, eugenol-induced anesthesia was the fastest, with a mean value of 118 ± 17.2 s, followed by buffered MS-222, with a mean value of 163 ± 33.2 s. Non-buffered MS-222, with a mean time of 185 ± 36.5 s, required the longest time to achieve the desired anesthetic state in the study. Finally, no interactions between effects were significant (anesthetic agent*sex, *p* = 0.76; anesthetic agent*exposure, *p* = 0.88; anesthetic agent*exposure*sex, *p* = 0.80).

### 3.2. Recovery from Anesthesia

**Figure 3:**
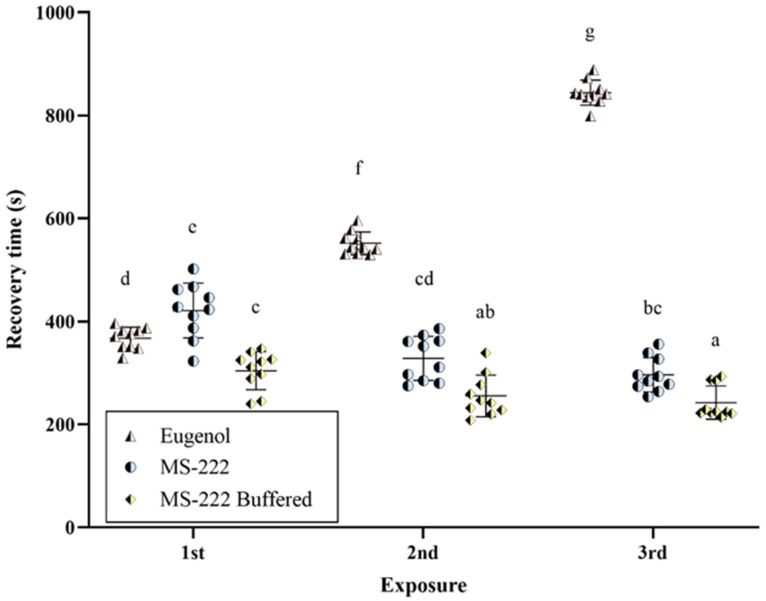
Recovery times for each zebrafish exposed to each anesthetic agent after three exposures. The lines and bars represent the mean and the standard deviation, respectively. Different letters indicate statistically significant differences (*p* < 0.05).

Similar to the induction of anesthesia, the random effect of each zebrafish (*p* = 0.13) and the fixed effect of sex (*p* = 0.81) did not show statistical significance in the time that animals need to recover. In addition, it was found that the anesthetic agent used also had significance (*p* < 0.05), the same as in the induction. The post hoc analysis revealed significant differences between the three anesthetic agents. The order of recovery times from anesthesia was as follows: MS-222 buffered < MS-222 < eugenol, with mean values of 267 ± 44.6 < 348 ± 68.4 < 587 ± 200 s, respectively. Furthermore, the interaction between the anesthetic agent and the exposure was also significant (*p* < 0.01). In general, MS-222 and MS-222 buffered exhibited shorter recovery times after the first exposure, while the opposite was observed with eugenol. The grouping of anesthetic agents based on exposure and recovery time is shown in Figure 3. Finally, the interactions between anesthetic agent*sex and anesthetic agent*sex*exposure were not significant (*p* = 0.39 and *p* = 0.21, respectively).

### 3.3. Post-Anesthesia Behaviour

**Figure 4:**
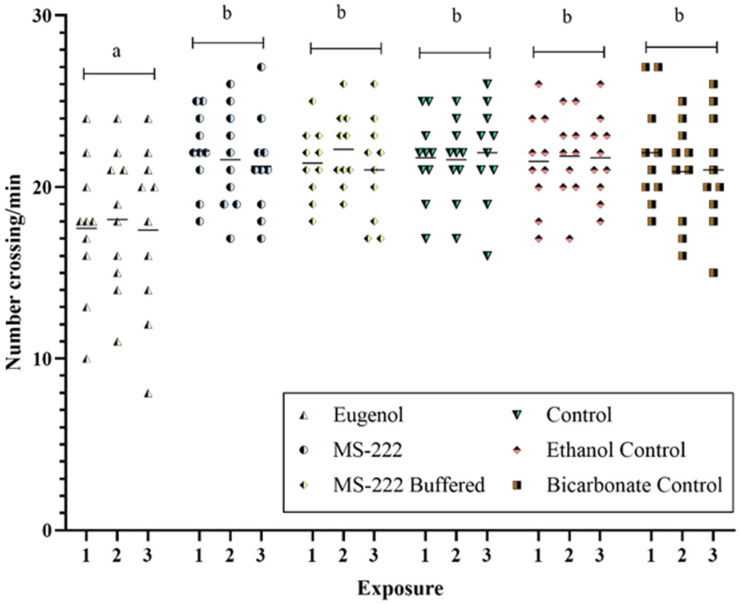
Frequency of crossing the aquarium’s longitudinal line per minute (crossings/min) at 23 h after repeated (1st, 2nd, 3rd exposure) anesthesia for each zebrafish. The lines represent the mean. Different letters indicate statistically significant differences (*p* < 0.05).

During the post-anesthesia observation period, no deaths were recorded in any of the groups exposed. It should be noted that the zebrafish anaesthetized with eugenol displayed lesser activity (*p* < 0.05) than those with MS-222, both the buffered and non-buffered solutions, the same as the three control groups (control, ethanol control, and bicarbonate control). Regarding this, Figure 4 shows the activity of the zebrafish determined in terms of the frequency of crossing the longitudinal line of the aquarium per min (crossings/min) at 23 h after anesthesia in the repeated exposures. Except for the fixed effect of the anesthetic agents, including the control groups, no other fixed factor, random effect, or interactions were statistically significant.

## 4. Discussion

Few studies have been performed regarding repeated anesthesia in adult zebrafish, which is very relevant. This relevance lies in common practices that are carried out during investigations. For example, a common procedure in zebrafish that requires repeated anesthesia is repeated blood extraction [34]. Blood is fundamental in animal research due to its high capacity to provide results. Currently, another common practice where successive anesthesia is required is the creation of pathologies in zebrafish [35]. Focusing on this relevance, we have observed that although eugenol is more potent to induce anesthesia than MS-222, it could depress the swimming activity in zebrafish after anesthesia. On the other hand, while it is internationally acknowledged that the utilization of a buffer for MS-222 is consistently advocated, there exists no substantiated evidence concerning the recurrent application of this substance as an anesthetic agent without buffering. Such a practice could conceivably give rise to deleterious consequences or alterations in the behaviour of zebrafish. Notwithstanding that the behaviour at the 23-h mark remains unaffected, prolonged induction and recuperation durations have been noted, underscoring the heightened necessity for the incorporation of a buffer.

With respect to the concentrations of the anesthetics given in the study, the concentration of 150 mg/L coincides with that cited by a large number of authors to induce light anesthesia in zebrafish [9,26,29,36]. It should be noted that Chambel et al. [29] detected mortality using the dose of MS-222 employed in this study. However, the anesthesia duration used for these authors was 30 min. In addition, Readman et al. [15] used a dose of 100 mg/L to achieve stage V, although in a different zebrafish strain (WIK strain).

Regarding eugenol, it is not the same as clove oil, although several articles refer to it as it was. In this context, authors have reported that concentrations comprised between 60 and 140 mg/L are necessary to reach stage V in that animal [26]. On the other hand, it has also been indicated that a concentration of between 60–100 mg/L is sufficient for attaining surgical anesthesia (stage VI) [9]. As can be observed, the effective concentration of eugenol at the different anesthesia stages is not well defined in the zebrafish, but that is not the case for MS-222. This can be explained because MS-222 is the most used anesthetic in aquaculture and research [18], and its use is allowed in fish destined for human consumption with a withdrawal period of 21 days before slaughter [37], while the Food and Drug Administration (FDA) and European Union do not authorize eugenol as an anesthetic for this purpose [38,39]. In this regard, the absence of a clear description of the anesthesia stage, the lack of research on repeated anesthesia use, and an effective dosage for the latter could have a negative impact on animal welfare [11].

The results obtained in our study for eugenol coincide with those described by other authors. Namely, eugenol has shorter induction times and provides a consistent and reliable state of anesthesia in zebrafish compared with others, such as ketamine. However, its recovery times are more prolonged [40,41,42], and, in addition, it can be observed how, throughout the repeated exposures, this time augments, probably due to its lipophilic character [43]. It should be noted that the use of eugenol involves the use of ethanol as a solvent, which could influence the anesthesia. However, authors have also reported that ethanol concentrations causing harmful effects in zebrafish are much higher than those used in this study. For example, Gerlai et al. [44] exposed zebrafish to a concentration of 0.25%, 0.5%, and 1%; 1% is equal to 7890 mg of ethanol for one hour, which is a much longer duration and a higher dose compared to ours, and they found no abnormalities in the posture of the fish or motor patterns. Moreover, Agues-Barbosa et al. [45], who exposed zebrafish to a concentration of ethanol of 0.25% for 30 min, observed an excitatory effect on locomotion. Therefore, the impact of ethanol in this study would be negligible.

Regarding the MS-222, its induction times scarcely displayed any significant differences in the successive exposures. However, the subsequent recovery times were shorter than those recorded in the first exposure to that compound. After successive exposure to MS-222, no increases in induction times have been seen in the Atlantic salmon (*Salmo salar*) either [46]. Nevertheless, it has also been observed that the induction time after repeated exposures to MS-222 increases in juvenile angelfish (*Pterophyllum scalare*) [47] or decreases in hybrid tilapia (*Oreochromis niloticus* × *Oreochromis aureus*) [48]. Therefore, it is suggested that it could depend on the species. To explain the short recovery in the zebrafish anaesthetized with MS-222 in general, its inhibitory effect on the nervous system is conditioned by its rapid diffusion through the gills [14,49]. Because of the latter and their fast metabolism, the concentrations in blood would diminish if the doses used were not high enough to exceed the velocity at which the active form of the drug is excreted from the organism [49]. Regarding the reduction in recovery time after the first exposure, perhaps specific physiological factor of the species that has not been considered could explain it.

The reduction of the induction and recovery times of buffered MS-222, with respect to non-buffered MS-222, could be related to the decline in the potency of the MS-222 when reducing the pH [50]. The pKa of MS-222 is about 3.5, which makes it a strong acid [51]; therefore, in more acidic water, i.e., in non-buffered water, the molecule of MS-222 tends to donate protons (H^+^) more readily, making it exist predominantly in its protonated and ionized form. In this regard, the ionized form of MS-222 is less effective at crossing biological membranes, such as the gill surface [32,52]. In general, the anesthesia aquarium water should be maintained with a stable pH close to neutrality (pH = 7) since the water’s pH could have negative effects on the health of the zebrafish. Additionally, sudden changes in the pH could be even more harmful [53]. In fact, when employing MS-222, it is recommended to apply buffer solutions since it is generally acquired in an acidic form, with a pH as low as 2.8 [16]. Some anesthetic concentrations with MS-222 reached a pH of 6.4, as we indicated in the Materials and Methods.

In our study, we did not observe any rise in mortality linked to the repeated use of MS-222, an occurrence that has been documented in zebrafish embryos and larvae [54]. Regarding post-anesthesia behavior, fish exposed to eugenol displayed a decrease in vertical activity within the water column when compared to those exposed to MS-222. Both eugenol and MS-222 are absorbed via the gills and skin [14,21]. Nevertheless, their molecular structures result in distinct behaviors. Eugenol, for instance, is lipophilic, whereas MS-222 is water-soluble. Consequently, eugenol exhibits a prolonged half-life, e.g., 12 h in rainbow trout (*Oncorhynchus mykiss*) [43], while MS-222 has a shorter half-life. Presently, there are no established half-life values for MS-222 in zebrafish, but values have been determined in other species, such as 5.27 h in barramundi (*Lates calcarifer*) (30 mg/L MS-222) [55], 1.7 min in Atlantic salmon (*Salmo salar*) (60 mg/L MS-222) [56], or 56 min in spiny dogfish (*Squalus acanthias*) [51]. In fact, MS-222 is considered a single-compartment anesthetic because its elimination is proportional to its plasma concentration [47].

Furthermore, their mechanisms of action also differ. MS-222’s mechanism of action is primarily attributed to the blocking of sodium channels in muscle tissue, with a lesser effect on potassium channels in nerve membranes [7]. This anesthetic does not interfere with the HPI (hypothalamus-pituitary-interrenal) axis, which is crucial when selecting an anesthetic for zebrafish, as it prevents alteration of the stress response during experiments [57,58]. The MS-222’s mechanism of action may be less harmful than that of eugenol, which is linked to its impact on ion channels in neuronal cells involved in nociception, neuronal peak generation, and synaptic transmission. Eugenol is a recognized inhibitor of the NMDA receptor of glutamate and an agonist of the neurotransmitter (GABA) [59]. These pharmacodynamic and mechanistic distinctions between the two drugs could elucidate the observed trends of increased recovery time with each exposure and the reduction in swimming frequency [32,37].

Particularly, it is suggested that the fact that eugenol is an agonist of GABA, the main inhibitory neurotransmitter of brain activity, and its prolonged time in the organism may be the cause of our results. Other variables, including exposure duration, anesthetic concentration, and species-specific characteristics (such as size, metabolic rate, and health status), have been excluded as significant random effects due to the uniformity of dosing, selected time intervals, and the subjects exposed in our study.

Considering the findings obtained in this study, it is suggested that eugenol could not be the most appropriate anesthetic in research requiring procedures that need successive anesthesia, such as obtaining blood samples or administering drugs multiple times, as it could negatively impact the experimental results. Additionally, a single exposure to eugenol has already reduced swimming frequency after several hours, which could also compromise the validity of the studies. This decrease in locomotor activity could have significant implications for the behaviour and welfare of the fish, as a longer recovery period could cause stress and affect their health. This study only determined swimming frequency, a reliable behavioral indicator; however, other more subtle adverse effects may not have been detected. Therefore, research on fish anesthesia must continue to clarify these aspects.

## 5. Conclusions

Based on the outcomes of this investigation, to achieve stage V anesthesia in zebrafish through a 10-min exposure, notable increases in recovery duration and a decline in swimming frequency within the water column were observed in the case of eugenol when compared to both formulations of MS-222. Conversely, the incorporation of bicarbonate to buffer the acidic properties of MS-222 enhances its suitability for recurrent anesthetic use, thereby further justifying its adoption.

It remains true that the experimental design of a study can exhibit significant variations, especially in scenarios where serial sample collection is imperative, necessitating multiple anesthesia administrations within a brief timeframe. Nevertheless, based on our results, MS-222 appears to be the preferable choice. However, it is important to emphasize that more research is needed to ensure the safety of one anesthetic agent over another. Regarding this last point, it is crucial to underscore that no flawless anesthesia protocol exists at present, and continued research in this domain remains indispensable. Future investigations, encompassing diverse concentrations, alternative pharmaceutical agents, and varying exposure durations, among other factors, will contribute to the ongoing enhancement and refinement of zebrafish anesthesia protocols.

## Data Availability

The data that support the findings of this study are available on request from the corresponding author.
